# Achievement Motivation and Performance in Wargames: Creativity as a Mediator

**DOI:** 10.3390/bs15040557

**Published:** 2025-04-21

**Authors:** Weiwei Xu, Sihui Ge, Dang Ding, Xiaopeng Ren

**Affiliations:** 1 Institute of Psychology, Chinese Academy of Sciences, Beijing 100101, China; xuww@psych.ac.cn (W.X.); gesh@psych.ac.cn (S.G.); dingd@psych.ac.cn (D.D.); 2 Department of Psychology, University of Chinese Academy of Sciences, Beijing 100101, China

**Keywords:** wargame, achievement motivation, creativity, performance

## Abstract

Computer-based wargames provide an experimental platform for studying cognitive antecedents and behavioral outcomes in dynamic scenarios. Our study examines how achievement motivation influence wargame players’ performance through the mechanism of creativity. In Study 1, we simplified the achievement motivation scale and revised the creativity scale for wargame contexts in China. After collecting data from students and wargame players (N1 = 300, N2 = 347), we validate their reliability and validity using exploratory and confirmatory factor analyses. Study 2 (N3 = 171) applied these validated scales to analyze the mechanism of creativity between achievement motivation and wargame performance. The results in Study 1 demonstrated that the refined two scales exhibited strong reliability and structural validity. The findings of Study 2 revealed that two types of motivation had different influences on wargame performance. The motivation of hope of success indirectly enhanced wargame performance through increased creativity. In contrast, the motivation of fear of failure reduced creativity and then negatively influenced overall results. Our study advances understanding of achievement motivation in dynamic gaming environments, suggesting that enhancing motivation of hope of success, decreasing motivation of fear of failure, and improving creativity may optimize performance to be more effective.

## 1. Introduction

Wargames refer to simulation-based strategy games involving a combination of maps, rules, and scenarios (Gao et al., 2022). The origins of wargames can be traced back to ancient strategic board games, such as chaturanga from sixth-century Ancient India and Chinese chess. Computer-based wargames enable the recreation of complex real-world combat situations without being constrained by physical space or material limitations (Mittal & Davidson, 2020). Computer-based wargames have become an important tool in the fields of education, military training, and entertainment due to their strategic and immersive nature (Hirst, 2022; Reinecke, 2009; Rosen & Kerr, 2024). Computer-based wargames are characterized by dynamic decision-making environments, information uncertainty, and resource constraints (Caffrey, 2019; Crookall, 2010; Perla, 1990). Good performance in these games depends on the effective implementation of motivation and creative problem-solving (Deci & Ryan, 2000; Locke & Latham, 2002). Wargaming, as a unique genre of strategy games, is distinct from other game types such as role-playing games or action games. Its core feature lies in its high-fidelity simulation of real-world war scenarios, emphasizing strategic planning, resource management, and decision-making rather than mere entertainment or reaction speed (Perla, 1990). This characteristic not only makes wargaming a recreational tool but also a critical instrument for military training and strategic research.

The distinction between online and offline gaming further underscores the representativeness of wargaming. Compared with offline games, online games offer greater flexibility and scalability, enabling large-scale data collection and diverse experimental designs (Yee, 2006b). For instance, online wargaming in laboratory projects can support both player-versus-player and player-versus-computer interactions (Hodicky & Hernandez, 2021). Moreover, the widespread popularity of online gaming provides a vast sample base for research. According to recent statistics, the MiaoSuan wargame used in our current research boasts over 50,000 players, offering extensive data support and practical significance for studies based on wargaming (Xu et al., 2022).

In recent years, the study of gaming motivation has attracted widespread attention, with many researchers being dedicated to developing and validating various gaming motivation scales. From earlier scales such as the GAMS (gaming motivation scale) and DGMS (digital game motivation scale) to more recent developments like the MOPS (motivation to play scale) and PMPVGS (psychological motivations for playing video games scale), these tools continue to be actively studied and applied in different research contexts. Notably, many of these scales include ‘achievement’ as a core motivational dimension, highlighting its importance in understanding gaming behavior. For instance, the GAMS, based on self-determination theory, indirectly reflects the impact of achievement motivation by assessing intrinsic motivation, integrated regulation, identified regulation, introjected regulation, external regulation, and amotivation (Lafrenière et al., 2012). The DGMS evaluates achievement motivation in gaming through eight motivational dimensions, including performance and agency (De Grove et al., 2016). While previous scales have focused on specific theories, the newer MOPS is a more comprehensive assessment of players’ general motivation to play games and incorporates 10 factors that include motivations such as creativity, autonomy, and achievement (Holl et al., 2024). The PMPVGsfurther proposes a sixteen-factor model that also includes competition, as well as escapism, completionism, and other important motivations, providing a detailed exploration of the different aspects of players’ motivations in games (Gursesli et al., 2023). These studies indicate that achievement motivation holds a significant position in the understanding of gaming motivation, with different scales employing various methods and dimensions to assess it.

Classical achievement motivation theory (Atkinson, 1957) proposes that the pursuit of success (hope of success, HS) and the avoidance of failure (fear of failure, FF), as a two-dimensional structure of motivation, are the core antecedent variables explaining individual performance differentiation through task choice, risk preference, and cognitive engagement. The HS’s tendency to converge may be invalidated by high-risk attributes, whereas the FF’s avoidance strategy may be subject to effects due to imbalances in the allocation of cognitive resources (Baumeister et al., 2007; Kahneman, 2011). However, although previous studies on classical achievement motivation theory have focused on academic performance (Amrai et al., 2011) or organizational management (Lee & Liu, 2009), prior studies have not fully explored motivational factors in computer-based decision-making tasks, such as wargaming. Wargaming is characterized by tasks that may reconfigure the pathways of motivation and performance because it is a complex system combining high uncertainty, resource constraints, and multi-round strategy iterations (Perla, 1990). This potential relationship also suggests that it is important to apply classical achievement motivation theory in computer-based games to explore its motivational factors.

Creativity has been shown to have predictive power for performance because it tends to enhance competency in complex problem-solving (Gajda et al., 2017) and innovative performance (Amabile, 2018). However, its role in dynamic adversarial tasks, such as wargames, remains controversial. On the one hand, the formal rules and constraints of wargames may inhibit unconventional creative thinking (Perla, 1990). On the other hand, its flexible and strategic decision-making framework provides room for tactical innovation (Mintzberg, 1994; R. Smith, 2012). The impact of creativity on performance is context-dependent: it may either enhance performance through breakthrough strategies or decrease performance by increasing the cost of risk (A. M. Grant & Berry, 2011; Zhang & Bartol, 2010a). However, the existing literature has not systematically examined the mediating mechanisms of creativity within the ‘motivation-performance’ pathway, particularly in the context of wargame.

The concept of creativity has been extended to firms and organizations, leading to the development of the construct of creativity (Zhou & Shalley, 2003), which emphasizes the contextual manifestation of creativity. However, there is a lack of measurement tools for creativity within the wargame context. Despite the relative maturity of instruments for measuring achievement motivation (Atkinson, 1957; Gjesme & Nygard, 1970) and creativity (Amabile, 2018; Zhou & George, 2001), traditional scales often involve lengthy questions. Lengthy questionnaires may lead to fatigue-induced satisficing (e.g., selecting the first plausible answer), resulting in measurement errors and reduced data quality (Krosnick, 1991). This can interfere with task progression, ultimately affecting the validity of the measurements (Dillman et al., 2014; Krosnick, 1991; Podsakoff et al., 2003). Thus, this study aims to refine the existing creativity scale and simplify the questionnaire to better suit this specific context in China.

By adapting and simplifying the scale in our Study 1, we revised two measurement tools for the context of wargames. Based on the adapted scale, Study 2 examined the different effects of achievement motivation of HS and FF on performance within the context of wargames. This will offer new theoretical perspectives and practical insights for predicting behavior in computer-based task environments. First, this study expands achievement motivation theory by explaining a dynamic ‘motivation-creativity-performance’ model. Second, this will contribute to theoretical advancements in predicting individual behaviors by motivational and capable factors in complex task environments. Third, our study could provide empirical evidence for optimizing training and improve people’s performance in computer-based tasks by increasing motivation and creativity.

## 2. Literature Review

### 2.1. The Relationship Betweeen Achievement Motivation and Wargames Performance

#### 2.1.1. Motivation Theory

Atkinson’s (1957) achievement motivation theory posits that achievement-related actions arise from the dynamic interplay between two opposing motivational forces: the HS and the FF. HS is a dispositional tendency to experience pride in accomplishment, driving individuals to seek out and engage in tasks offering opportunities for mastery. FF is a predisposition to experience shame upon failure, motivating avoidance of tasks where failure is perceived as probable or consequential. Building on Atkinson’s (1957) work, McClelland (1985) proposed that individual behavior is driven by three core needs: achievement, affiliation, and power, with the need for achievement being particularly influential in goal-directed contexts. Higgins’ regulatory focus theory (1997) further differentiated between promotion focus (pursuing success) and prevention focus (avoiding failure), revealing how these orientations shape creativity and goal attainment. Meanwhile, Elliot’s achievement goal theory (Elliot & McGregor, 2001) linked specific goal types (e.g., mastery-approach) to performance outcomes. Together, these theories—from McClelland’s needs to Higgins’ regulatory foci and Elliot’s goal frameworks—provide a comprehensive lens for understanding motivation in complex, uncertain environments.

Additionally, Yee (2006a) proposed a motivation model for online games that includes three dimensions: achievement, social interaction, and immersion. In the achievement dimension, Yee emphasized that players pursue progress (e.g., character leveling and task completion), understand game mechanics (e.g., strategy and skill mastery), and compete (e.g., rankings and victories in PVP battles). This model reveals how players balance and interact between these motivational dimensions in their pursuit of game achievements.

Based on the achievement motivation theory, Norwegian psychologists Gjesme and Nygard (1970) developed the achievement motivation scale (AMS) in the 1970s, which was specifically designed to measure the bidimensional constructs of HS and FF. The scale consists of 30 items, divided into two subscales. First, the HS subscale (15 items), which assesses an individual’s tendency to actively seek out challenges and pursue excellence. Secondly, the FF subscale (15 items), which measures an individual’s sensitivity to and avoidance of the risk of failure. The scale utilizes a four-point Likert-type scale (1 = strongly disagree, 4 = strongly agree) and reflects the individual’s overall level of achievement motivation by calculating the difference between the HS and FF scores (HS-FF).

The Chinese version of the AMS was first introduced and adapted by Ye (1992) to better suit the Chinese cultural context. The revised scale demonstrated strong reliability and validity within groups of college and middle school students: α = 0.81 for the HS subscale and α = 0.87 for the FF subscale (J. Wang & Zheng, 2010). Its bidimensional structural stability aligns with that of the HS and FF scores. The stability of its bidimensional structure and practicality has led to its widespread use in the fields of educational psychology and organizational behavior in China, such as predicting academic performance, performance, and innovative behavior (D. Chen et al., 2020; L. Chen et al., 2017).

#### 2.1.2. Achievement Motivation and Performance

Achievement motivation theory (Atkinson, 1957) suggests that an individual’s performance is driven by two core motivations: HS and FF, which shape goal-directed behavior (Elliot & McGregor, 2001; Higgins, 1997). Research indicates that HS promotes performance by enhancing goal commitment, with significant findings in academic contexts, where mastery goals linked to HS predict higher academic performance and engagement (McClelland, 1985). In sports, HS-driven athletes tend to set higher goals and show greater persistence, leading to improved performance (R. E. Smith et al., 2009). Similarly, in gamification, players with high HS engage in creative strategies that enhance game performance (Sailer et al., 2017; Song et al., 2013) (Sailer et al., 2017; Song et al., 2013). Thus, we predict:

**Hypothesis** **1.** *Hope of success has a positive impact on the performance.*

In recent years, more evidence suggests that FF may enhance performance by stimulating risk aversion and fine-tuning strategy choices in specific contexts (Conroy & Elliot, 2004). For example, it was found that FF-driven individuals tended to invest more cognitive resources in high-risk tasks to avoid potential losses (De Castella et al., 2013; Sweller, 1988). Individuals with FF are more likely to pursue perfectionism, which can sometimes enhance their engagement in tasks (Hewitt & Flett, 1991). Thus, FF improved the quality of task completion. Regulatory focus theory incorporates FF into a prevention focus framework (Higgins, 1997), suggesting that FF optimizes the robustness of performance by strengthening responsibility awareness and risk anticipation. Therefore, we propose the following hypothesis:

**Hypothesis** **2.** *Fear of failure has a positive impact on performance.*

### 2.2. The Mediating Role of Creativity

#### 2.2.1. Creativity Theory

Creativity is usually defined as an individual’s ability to generate novel and useful ideas, products, or solutions (Amabile, 2018). In the field of computer-based behavior, creativity refers to the ability to come up with innovative ideas or solutions in work situations (Zhou & Shalley, 2024). Compared with general creativity, creativity in computer-based context emphasizes task relevance and organizational adaptability, i.e., innovations need to serve organizational goals and have practical applications (Oldham & Cummings, 1996). Because creativity places more emphasis on innovative behaviors in organizational contexts and its measurement tools have been widely used in management and psychological research (Gong et al., 2009), this study revised creativity as the core measurement concept in the context of wargames.

The creativity scale (Zhou & George, 2001) has been adapted in the Chinese cultural and organizational context and has demonstrated high reliability and validity in organizational behavior research. The scale was designed to measure subjects’ creativity in specific contexts, especially in the work environment. Studies have shown that the scale has a Cronbach’s alpha of 0.94 in the Chinese setting (Y. Wang et al., 2012). In addition, Pan et al. (2013) also verified the applicability of the scale, reporting a Cronbach’s alpha value of 0.91.

#### 2.2.2. Self-Determination Theory

Self-determination theory (SDT) provides an important theoretical framework for understanding the relationship between motivation and creativity. According to SDT, motivation influences creativity through three main mechanisms. First, intrinsic motivation significantly enhances creative performance by stimulating an individual’s interest and autonomy in the task itself (Deci & Ryan, 1985). Second, extrinsic motivation can positively predict creativity only when it is highly internalized (e.g., integrated regulation aligning with personal values). Finally, motivation influences creativity through the satisfaction of basic psychological needs (e.g., autonomy, competence, and sense of belonging) (Przybylski et al., 2010).

However, despite the extensive validation of these mechanisms in offline gaming environments, significant gaps remain in their applicability to online gaming contexts (Hainey et al., 2011). Prior research has failed to adequately explain how online play influences creativity through motivation and how creativity further mediates the relationship between motivation and game performance (Hulaj et al., 2020; Mun, 2022). Based on this, this paper aims to explore the role of creativity as a mediating variable in online gaming from the perspective of self-determination theory, revealing the complete pathway from motivation to game performance. This study not only bridges the gap in the existing literature but also provides a new theoretical perspective for understanding the dynamic relationship between motivation and creativity in online gaming environments.

Higgins’ (1997) regulatory focus theory posits that individuals’ goal pursuit is guided by two distinct motivational orientations: promotion focus (striving for gains, aspirations, and achievement) and prevention focus (avoiding losses, obligations, and failure). Promotion-focused individuals prioritize innovation and risk-taking to maximize rewards, while prevention-focused individuals adopt conservative strategies to minimize errors (Higgins, 2000). These orientations align closely with the dual dimensions of achievement motivation: HS and FF (Elliot & Harackiewicz, 1996). Empirical studies demonstrate that promotion-focused motivation fosters creativity by encouraging exploratory thinking and novel problem-solving (Friedman & Förster, 2001). For instance, in complex decision-making tasks, promotion-focused individuals exhibit higher creative flexibility, which directly enhances performance outcomes (Crowe & Higgins, 1997). Conversely, prevention-focused motivation, driven by FF, often correlates with rigid cognitive patterns and risk aversion, potentially stifling creative processes (Roskes et al., 2012).

In strategic gaming contexts like wargames, creativity is a critical determinant of performance, as players must rapidly generate unconventional tactics under uncertainty. One study in strategic video game shows that higher self-reported problem-solving skills predicted higher academic grades (Adachi & Willoughby, 2013). Research on competitive gaming reveals that promotion-driven players outperform peers in dynamic environments by leveraging creative strategies (Adachi & Willoughby, 2013). For example, chess players will prioritize defensive moves over innovative moves due to cognitive fixation, leading to suboptimal results (Bilalić et al., 2008). It is possible that FF may inhibit exploratory behaviors, which indirectly impair performance (Elliot & Church, 1997). Notably, creativity’s mediating role in this relationship remains under-explored. While promotion focus may enhance performance through creativity, prevention focus could paradoxically reduce creativity’s utility (Higgins, 2000; Zhou, 1998).

Previous studies suggest a positive relationship between HS and creativity. For example, Snyder (2002) proposed that individuals with high HS exhibit greater cognitive flexibility and problem-solving abilities, which are critical components of creativity. This aligns with Amabile’s (2018) intrinsic motivation theory, which posits that goal-oriented motivations foster divergent thinking by encouraging individuals to explore novel solutions. Additionally, Dweck (2006) noted that a growth mindset—closely linked to HS—promotes resilience in creative tasks, as individuals view challenges as opportunities rather than threats (Dweck, 2002; Dweck et al., 2014). Building on these findings, we propose that creativity acts as a *positive mediator* in translating HS into enhanced wargame performance. Creative strategies generated through goal-directed motivation (Snyder, 2002) may enable players to adapt dynamically to complex scenarios, thereby improving tactical outcomes (Ford & Gioia, 2000). This mediation pathway is further supported by studies linking creativity to performance in strategic games (Gong et al., 2013).

The influence of creativity on performance has been extensively validated. A study by George and Zhou (2002) demonstrated that positive emotions stimulate creativity, which in turn generally leads to higher job performance. This finding supports the positive association between creativity and job performance. Imran et al. (2018) further indicated that creativity plays a mediating role in facilitating knowledge processes and enhancing firm performance. The study highlighted that creativity indirectly contributes to performance by enhancing efficiency and effectiveness. Additionally, there exists an interaction between task complexity and creativity, which significantly influences performance. Sung et al. (2017) found that the complexity of work tasks can stimulate employees’ creative problem-solving abilities, thereby enhancing performance. Particularly, the role of creativity is more pronounced in high-complexity tasks, where employees can effectively address complex challenges, thereby demonstrating superior performance. An empirical study by Zhang and Bartol (2010b) further emphasizes the positive influence of creative process inputs on performance, suggesting that this effect follows a curvilinear pattern. Excessive creative input may lead to fatigue or resource depletion, whereas moderate input maximizes performance outcomes. Creativity has been demonstrated to significantly predict individual and team performance in high-complexity tasks and dynamic environments. Shalley et al. (2004) demonstrated that creativity can effectively address highly uncertain task environments and enhance team performance by fostering innovation and problem-solving. Another research also found that creativity indirectly enhances team innovation performance by promoting knowledge-sharing and problem-solving (Gong et al. (2009)). Zhou and Shalley (2024) further suggest that creativity is a critical competency for addressing complex challenges in high-uncertainty tasks.

The existing literature generally supports the view that creativity has a positive effect on performance. Based on the above theoretical and empirical studies, this study proposes the following hypothesis:

**Hypothesis** **3.** *Creativity mediates the relationship between hope of success and performance such that higher hope of success leads to higher creativity and then higher creativity leads to higher performance.*

In contrast, prior studies suggest a positive relationship between FF and creativity. For instance, Byron et al. (2010) found that FF can stimulate creative risk-taking in high-pressure contexts, as individuals attempt unconventional methods to avoid negative outcomes. Similarly, Roskes et al. (2012) argued that avoidance motivations may temporarily enhance creativity by narrowing focus on threat mitigation. However, this relationship may be context-dependent. While fear-driven creativity might generate short-term adaptive strategies, its long-term impact on performance could be detrimental. For example, Mueller et al. (2011) demonstrated that anxiety associated with FF often leads to cognitive rigidity over time, stifling effective innovation. Extending this logic, we hypothesize that creativity serves as a *negative mediator* between FF and wargame performance. Although FF may initially spark creative tactics, the accompanying stress and aversion to uncertainty (Elliot & Church, 1997) could undermine decision-making precision, ultimately reducing gameplay efficacy. This aligns with findings in competitive gaming literature, where excessive risk aversion correlates with suboptimal in-game choices (Holt & Laury, 2002).

**Hypothesis** **4.** *Creativity mediates the relationship between fear of failure and performance such that higher fear of failure leads to lower creativity and then lower creativity leads to lower performance.*

**Figure 1:**
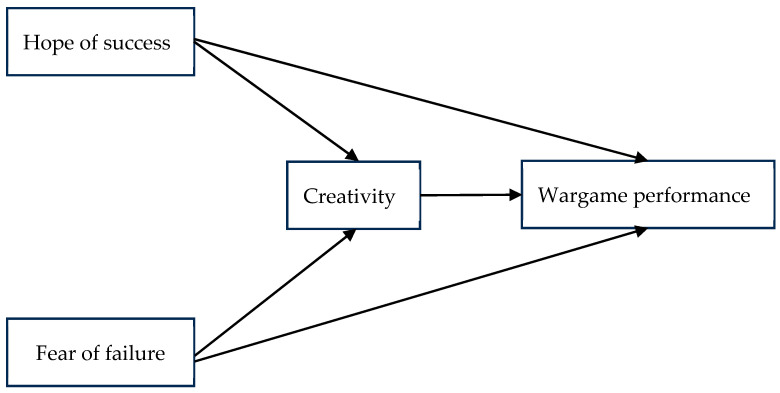
The proposed theoretical model.

In summary, we propose our theoretical model in Figure 1.

## 3. Study 1

### 3.1. Materials and Methods

#### 3.1.1. Participants

We collected data from university students and wargame players. In Sample 1, participants were recruited from universities in Beijing, and they all signed an informed consent form prior to completing an online questionnaire. A total of 307 questionnaires were distributed to participants. At the end of the screening process, questionnaires with a response time of less than 200 s, as well as those with a deviation of more than 3 standard deviations from the mean, were excluded. Ultimately, 300 valid questionnaires were retained. In Sample 2, wargame players from North China were recruited, and a paper-based questionnaire was distributed after obtaining their informed consent. A total of 435 questionnaires were distributed, and after excluding incomplete responses, 347 valid questionnaires were retained. According to Gorsuch (2014), the sample size for exploratory factor analysis (EFA) should be 5 to 10 times the number of variables. Additionally, Hair et al. (2019) recommend a minimum of 10 participants per variable and a total sample size of at least 200 for robust results. For confirmatory factor analysis (CFA), Kline (2023) suggests a minimum sample size of 200 participants.

#### 3.1.2. Instruments

We used the achievement motivation scale, revised by Ye (1992), which is based on Gjesme and Nygard (1970). The scale comprises 30 items, divided into two dimensions: Hope of Success and Fear of Failure. The scale uses a 4-point Likert-type scale (1 = strongly disagree, 4 = strongly agree).

The creativity scale developed by Zhou and George (2001) was employed. The scale comprises 13 items and uses a 5-point Likert-type scale (1 = strongly disagree, 5 = strongly agree).

#### 3.1.3. Procedure

First, we refined the creativity scale by modifying certain items to better align with the context of this study. After these modifications, we administered the revised questionnaire to Sample 1, whose participants completed the full version of the achievement motivation scale and the modified creativity scale. The validity of the revised questionnaire was verified using the responses from Sample 1.

Following this validation, we selected a subset of items to form a simplified version of the questionnaire, based on expert evaluations. Research indicates that at least 3 items are necessary to demonstrate the validity of the simplified scale (Clark & Watson, 2016). The chosen items were carefully evaluated and selected by experts to ensure alignment with the objectives of this study. Subsequently, a statistical analysis was conducted between the full version and the simplified version using the data from Sample 1.

Next, the simplified questionnaire version was distributed to Sample 2, which consisted of wargame players. We used the questionnaire to further validate this group, ultimately confirming the effectiveness of the simplified version.

#### 3.1.4. Statistical Analysis

We conducted statistical analyses using SPSS 27.0 and AMOS 27.0 software. Sample 1 was tested via exploratory factor analysis (EFA) and Sample 2 was assessed through confirmatory factor analysis (CFA). EFA was used to examine the underlying relationship and to determine whether two or more variables could be combined into the same factor if they show a high correlation and share some common characteristics; meanwhile, CFA was used to confirm the factor structure identified via the EFA. The following model fit indices were selected: (1) χ^2^/df (chi-square to degrees of freedom ratio), where in some cases (e.g., with a large sample size), a χ^2^/df < 5 is also acceptable (Kline, 2023); (2) GFI (goodness of fit index), CFI (comparative fit index), TLI (Tucker–Lewis index), and IFI (incremental fit index), where values ≥ 0.90 suggest a good fit and values ≥ 0.95 indicate an excellent fit; (3) RMR (root mean square residual), where an RMR < 0.05 denotes a good model fit (L. T. Hu & Bentler, 1999); (4) RMSEA (root mean square error of approximation), where RMSEA ≤ 0.10 indicates that the fits are reasonable (Browne & Cudeck, 1992). A Cronbach’s alpha coefficient ranging between 0.5 and 0.7 is considered acceptable in exploratory research, while higher alpha values (such as 0.7 or above) are generally regarded as more ideal.

### 3.2. Results

#### 3.2.1. Sample 1

##### Descriptive Statistical Analysis

In Sample 1 (*N*1 = 300), the gender distribution comprised 170 males (56.7%) and 130 females (43.3%). Regarding sibling status, 117 participants (39.0%) were only children, while 183 (61.0%) had siblings. The sample’s age distribution showed a mean of 21.48 years (SD = 3.43), ranging from 18 to 47 years. Educational attainment levels were distributed as follows: 28 participants (9.3%) completed high school or less, 255 (85.0%) held undergraduate degrees, and 17 (5.7%) possessed graduate degrees.

##### Exploratory Factor Analysis

To verify the suitability of the data for factor analysis, this study conducted the Kaiser–Meyer–Olkin (KMO) measure of sampling adequacy and Bartlett’s test of sphericity on the original questionnaire data. The KMO value indicates whether there are sufficient common factors among the variables. A KMO value greater than 0.9 generally suggests that the sample is highly suitable for an exploratory factor analysis. The analysis results showed that the KMO value was 0.928 for the HS dimension, 0.944 for theFF dimension, and 0.928 for the creativity scale, all indicating that the data are appropriate for factor analysis. Concurrently, Bartlett’s test of sphericity assesses the suitability of data by determining whether the correlation matrix is an identity matrix. If the test result is significant (*p* < 0.05), it confirms that the sample data are suitable for a factor analysis. For specific results of Bartlett’s test of sphericity, see Table 1.

##### Reliability Testing

To assess the stability of the simplified scales, we conducted an analysis of homogeneity reliability. According to the analysis results, the Cronbach’s α coefficient was 0.643 for the simplified HS scale, 0.788 for the simplified FF scale, and 0.870 for the revised creativity scale. Additionally, internal consistency reliability analysis was performed to evaluate the measurement quality of each dimension’s items and their contribution to the overall scale. By calculating the Cronbach’s α values when items were deleted and the correlation coefficients between each item and the total score, the analysis assessed whether each item effectively reflected the core content of its respective dimension and verified the reliability of the scale.

The simplified HS scale contains three items. After the deletion of any item, the Cronbach’s α values ranged from 0.455 to 0.602, indicating high internal consistency within the scale. The correlation coefficients between each item and the total score ranged from 0.710 to 0.799, suggesting that the items significantly contribute to the total score. The simplified FF scale also contains three items. The Cronbach’s α values after item deletion ranged from 0.605 to 0.665, and the correlation coefficients between each item and the total score were high (0.831 to 0.848), reflecting high reliability and strong measurement validity of the items in this dimension. The two-factor model (HS/FF) of the achievement motivation scale explained 64.96% of the cumulative variance. Specific results are presented in Table 2.

The revised creativity scale includes four items. After item deletion, the Cronbach’s α values were all above 0.8 (0.807 to 0.852), and the correlation coefficients between each item and the total score ranged from 0.809 to 0.890, further demonstrating the high measurement consistency and item quality of the scale. The eigenvalue (eigenvalue) was 2.883, indicating that the extracted factor explained a larger proportion of the variance. The factor explained 72.080% of the total variance, indicating high structural validity of the scale. Specific results are presented in Table 3.

##### Validity Testing

We examined the correlation between the full scales and the simplified versions, revealing highly significant positive correlations (ranging from 0.858 to 0.947) between the full and simplified versions of each scale, which effectively reflect the measurement characteristics of the corresponding complete scales. Specific results are detailed in Table 4.

#### 3.2.2. Sample 2

##### Descriptive Statistical Analysis

The Sample 2 comprised 310 males (89.3%) and 37 females (10.7%). There were 145 only children (41.8%) and 202 non-only children (58.2%) in the sample. The average age of the participants was 23.75 ± 2.47 years, ranging from 20 to 47 years.

##### Confirmatory Factor Analysis (CFA)

We conducted a confirmatory factor analysis (CFA) on the questionnaire to assess the fit of the structural model and to evaluate the merits of the conceptual model. In the confirmatory factor analysis, the model fit indices are crucial for assessing the degree of congruence between the model and the data. The results of the confirmatory factor analysis are presented in Table 5. In the simplified achievement motivation scale and revised creativity scale, all fit indices met the recommended standards; χ^2^/df < 5; the values of GFI, CFI, TLI, and IFI are all close to 1; the RMSEA values were all below the threshold of 0.05; and the RMSEA values are all below 0.10.

##### Reliability Testing

We conducted consistency coefficient analysis on these scales. According to the analysis results, the Cronbach’s alpha coefficient for the simplified HS scale was 0.653. For the simplified FF scale, it was 0.727. And for the revised creativity scale, it was 0.899. These coefficients indicate the internal consistency of the items within each scale.

### 3.3. Discussion

In Study 1, the reliability of the revised creativity scale and simplified achievement motivation scale was rigorously examined. The exploratory factor analysis (EFA) demonstrated that the single-factor structure of the creativity scale accounted for 72.08% of the total variance, while the two-factor model (HS/FF) of the achievement motivation scale explained 64.96% of the cumulative variance. Confirmatory factor analysis (CFA) further validated the structural validity of both scales, with excellent model fit indices being observed. Robust positive correlations between the abbreviated and original versions provided strong evidence of convergent validity, confirming the simplified scales as psychometrically sound and efficient alternatives for future research.

By adapting and simplifying the scale in our Study 1, we revised two valid measurement tools for the context of wargame and the subsequent empirical analysis in Study 2. Accordingly, Study 2 examined the different effects of achievement motivation of HS and fear and failure on performance through the mechanism of creativity within the context of wargames.

## 4. Study 2

### 4.1. Materials and Methods

#### 4.1.1. Participants and Procedures

We conducted data collection for this study on the Wargame: *MiaoSuan* (http://wargame.ia.ac.cn/main (accessed on 19 April 2025)), developed by the Institute of Automation, Chinese Academy of Sciences. In the 2023 Human-AI Hybrid Competition, male players accounted for 90% of the participants. Therefore, to align with the competition’s demographics, this study selected male participants as subjects. This choice is also consistent with existing research, which has found that male players outnumber female players in online gaming (Hainey et al., 2011). We controlled for the participants’ gender, education level, and gaming experience, because previous research has demonstrated that these factors can influence the performance (Hainey et al., 2011). All participants recruited for this study were male first-year students from universities in Beijing who had never played wargames. A total of 180 participants signed the informed consent form, and all participants completed the simplified scale developed in Study 1. After excluding subjects with missing data during the competition, 171 participants with complete competition results and questionnaire data were included in the final analysis.

Before the competition, we trained the participants, explained the basic rules and operations of wargames, and watched teaching videos for 1 h. The experiment employed a paired competitive design, where each pair of participants engaged in four rounds of competition. Each participant alternated between playing as the red and blue sides, with each side being played for two rounds. Each round was divided into two phases: (a) deployment phase: lasting 300 steps, during which both sides performed their initial strategic deployments; (b) main competition phase: lasting 1800 steps, during which both sides engaged in formal combat. The game was conducted at double speed, with each round lasting approximately 30 min. Upon completion of each round, the battle data for each participant were downloaded through the management interface of the Wargame: *MiaoSuan* After summarizing the results of the 180 participants, those who had incomplete data due to early withdrawal were excluded, resulting in a final sample of 171 participants with valid data. We used the average score of 4 games played by each participant as a performance indicator. Performance metrics may adopt distinct operational definitions, such as win rate or in-game scores. Despite the fact that a high score cannot guarantee ultimate victory, it systematically correlates with superior in-game execution, thereby serving as a more nuanced proxy for strategic proficiency (Herold et al., 2021).

#### 4.1.2. Measurements

We utilized a simplified version of the measurement tool developed by Study 1. Internal consistency reliability analysis revealed that Cronbach’s α coefficients for each dimension were 0.513, 0.668, and 0.915, respectively. Notably, Cronbach’s α coefficients for achievement motivation and avoidance of failure motivation were slightly below the commonly recommended threshold of 0.7. However, according to Taber (2018) a Cronbach’s α coefficient of 0.5 or above is considered the minimum acceptable standard in exploratory research. Therefore, the measurement tools used in this study demonstrate reasonable reliability.

#### 4.1.3. Statistical Analysis

The statistical analysis included descriptive statistics, Spearman’s correlation analysis, regression analysis, and the bootstrap method to test for mediation effects. All analyses were conducted using SPSS version 27.0 and the AMOS.27.0 plugin to ensure the scientific rigor and reliability of the results.

### 4.2. Results

#### 4.2.1. Common Method Bias

Harman’s single-factor test was employed to assess common method bias. The results indicated that three factors had eigenvalues greater than 1. The variance explained by the first factor before rotation was 34.978%, which is below the critical threshold of 40%. This suggests that there is no significant common method bias among the variables in the study.

#### 4.2.2. Descriptives

The average age of the participants was 20.25 years (SD = 1.63), with an age range from 18 to 26 years. Of the sample, 71 participants (41.5%) were only children, while 100 participants (58.5%) were not. Table 6 presents the mean scores and standard deviations for creativity, HS, FF, and performance.

#### 4.2.3. Correlation Analysis

We conducted correlational analyses between the scores on various scales and performance, with specific results being presented in Table 6. There was a significant degree of correlation between the scores of the creativity, HS, and FF with performance. Specifically, creativity showed a significant positive correlation with the performance (r = 0.19, *p* < 0.05). The relationships between the scores on the creativity, HS, and FF suggest that creativity may have a positive influence on performance. The regression effect of HS on performance was not significant, which rejects Hypothesis 1. The regression effect of FF on performance was significantly positive, which supported Hypothesis 2.

#### 4.2.4. Mediation Analyses

To further explore the potential mediating effects and indirect influence of the independent variable on the dependent variable, we conducted mediation effect testing based on the bootstrap method to ensure the robustness and reliability of the results. Table 7 shows the specific mediating effects.

For the mediator of creativity between HS and performance, the total effect was not significant (estimate = 0.063, *p* = 0.435), with a 95% confidence interval of [−0.057, 0.153], suggesting that the overall effect of HS on creativity did not reach statistical significance. The direct effect was also not significant (estimate = −0.015, *p* = 0.792), with a confidence interval containing zero values (−0.119, 0.081), suggesting that the direct pathway may not be substantially related. The indirect effect was significant (estimate = 0.067, *p* = 0.002) with a confidence interval of [0.031, 0.117], suggesting that hope of success may indirectly affect creativity through the mediating variable and that the mediating effect was statistically significant. Our results supported Hypothesis 3.

**Figure 2:**
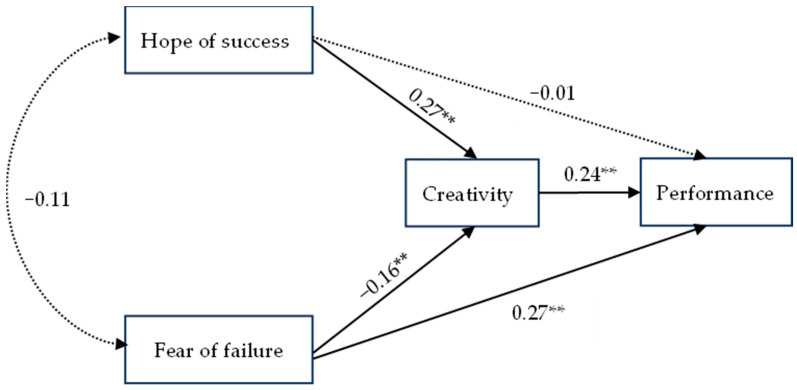
Mediation model and coefficients; ** *p* < 0.01.

For the mediator of creativity between FF and performance, the total effect was significantly positive (estimate = 0.071, *p* = 0.003) with a 95% confidence interval of [0.111, 0.344], indicating that the overall effect of FF on creativity was positive and significant. The direct effect was significantly positive (estimate = 0.274, *p* = 0.002) with a confidence interval that did not contain a zero value (0.144, 0.372), suggesting that there is an independent and direct contribution of FF to creativity. The indirect effect was significantly negative (estimate = −0.040, *p* = 0.012) with a confidence interval of [−0.087, −0.012], suggesting that there may be an inhibitory mediating pathway that partially counteracts the positive effect of the direct effect. The results supported Hypothesis 4. The model pathways are illustrated in Figure 2.

### 4.3. Discussion

The results of the correlation analysis showed a significant correlation between creativity and hope of success, FF, and performance. However, the correlation between HS and performance did not reach statistical significance, leading to the rejection of Hypothesis 1. Therefore, we cannot conclude that hope of success has a direct effect on performance. In contrast, the correlation between FF and performance was significantly positive, supporting Hypothesis 2 and indicating that FF has a positive predictive effect on performance.

Further mediation analyses revealed the mediating role of creativity between hope of success and performance. Although both the total and direct effects of hope of success on creativity were not significant, the indirect effect through creativity reached statistical significance, supporting Hypothesis 3. This suggests that hope of success may indirectly influence performance through creativity. The analysis of creativity as a mediating variable in the relationship between FF and performance showed that both the total and direct effects of FF on creativity were significantly positive, supporting Hypothesis 4. However, the indirect effect was significantly negative, suggesting the presence of an inhibitory mediating pathway that partially counteracts the positive direct effects. This finding provides new insights into how FF can have complex effects on performance through creativity.

## 5. General Discussion

To expand achievement motivation theory (Atkinson, 1957) by explaining the mechanism of creativity, this study empirically analyzed the effects of two core dimensions of achievement motivation in a wargaming environment: HS and FF on participants’ performance in wargame. HS had significantly positive relationship with performance through the mechanism of creativity. In contrast, FF had a significantly negative correlation with performance through the mechanism of creativity.

For the relationship between HS and performance, although the total effect and direct effect were not significant, creativity exerted a significant indirect effect. This suggests that HS may indirectly enhance performance by stimulating creative tactics, while the weakness of the direct pathway may stem from the inhibition of motivation by the wargame environment (Byron & Khazanchi, 2012). Amabile’s (2018) research confirms that when individuals are exploratory goal-oriented, achievement motivation influences performance by reconfiguring problem-solving strategies rather than directly acting on outcome indicators. This result supports the hypothesis that creativity serves as a key mechanism between achievement motivation and performance.

In contrast to FF, the direct predictive effect of HS on performance did not reach a significant level. Wargames are characterized by a temporal delay in evaluating the efficacy of tactical decisions, as the actual outcomes of strategic deployments in the first stage can only be fully assessed in the second stage. This finding may reflect performance—approach motivation may not enhance performance when clear incentives are lacking (Elliot & McGregor, 2001). This result is consistent with H. Grant and Dweck (2003), who found that the effect strength of HS is significantly moderated by task structure and reward accessibility. Because the computer-based wargame is characterized by dynamic decision-making environments, information uncertainty, and resource constraints (Caffrey, 2019; Crookall, 2010; Perla, 1990), it enables the FF-driven individuals to enhance decision-making efficacy through dynamic strategy adaptation, thus counteracting the negative impact of potential failure risk on performance (Carr & Steele, 2010). Moreover, prior research has demonstrated the predictive effect of FF on performance was significantly positive (Conroy & Elliot, 2004; Elliot & Thrash, 2004).

From a cultural perspective, the results of Study 2 are consistent with the findings of Chinese scholar Gan Yiqun on proactive and preventive coping. In the context of Chinese cultural values, the positive effects of wanting to succeed on creativity and performance are consistent with proactive coping, which emphasizes self-improvement and goal attainment values rooted in the Confucian ideal of striving for excellence (Gan et al., 2010) (Gan et al., 2010). In contrast, the negative effects of FF reflect preventive coping tendencies associated with risk aversion and maintaining harmony, another important Confucian principle (Y. Hu & Gan, 2011). These results suggest that Chinese cultural values may influence motivational dynamics, promoting proactive behavior in the HS and reinforcing a cautious approach in the FF. This cultural perspective provides a plausible explanation for the patterns observed in the Chinese sample.

In the relationship between FF and performance, creativity showed a significant negative mediating effect by suppressing creativity. Furthermore, FF decreased creativity, which is consistent with Higgins’s (1997) regulatory focus theory. Specifically, prevention-focused motivation (an avoidance-oriented dimension of the theory) may limit cognitive flexibility. While prevention-focused behavior enhances performance in the short term, its suppression of divergent thinking may reduce adaptability in the long term (Baas et al., 2011). In addition, the present study found that creativity was a significant positive predictor of player performance, which is consistent with the theoretical mechanism on creativity as a core competency in complex problem-solving. In conclusion, our study further revealed the mediation mechanism of creativity between achievement motivation and performance, supporting A. M. Grant and Berry (2011)’s proposed ‘motivation-creativity-performance’ chain model.

### 5.1. Theoretical Contributions and Empirical Implications

Our study extends achievement motivation theory (Atkinson, 1957) by explaining the mechanism of creativity between achievement motivation and computer-based wargame performance. First, this study expands achievement motivation theory by explaining the mechanism of creativity in a dynamic ‘motivation-creativity-performance’ model. Although previous research found mechanisms of risks and uncertainty explained the relationship between motivation and performance (March & Shapira, 1987; Sitkin & Pablo, 1992), our study considers a capable perspective of creativity to explain how two types of motivation influenced performance differently.

Second, this will advances behavioral prediction theory by integrating motivational and creativity factors in complex task environments. Many studies explored how different types of motivation affect performance (Deci & Ryan, 2013; Elliot & Harackiewicz, 1996). However, our study considered both motivational (i.e., HS and FF) and capable factors (i.e., creativity) that can influence performance at the same time. We suggest that increasing HS and FF could influence performance by affecting creativity in contrasting ways.

Third, our study could provide empirical evidence for optimizing training and improve people’s performance in computer-based tasks by increasing motivation and creativity. Although previous studies found that achievement motivation influenced performance in organizational performance (Locke & Latham, 2002) and academic performance, we used the computer-based tasks because it is increasingly common in daily work (Landers & Behrend, 2015) and in selecting job candidates (Tippins et al., 2006). Based on achievement motivation theory, our study reveals the pattern of differentiation of motivational effects in complex dynamic tasks, which compensates for the limitation of traditional research focusing on static situations.

### 5.2. Limitations and Future Work

Although our study expands achievement motivation theory (Atkinson, 1957) by explaining the mechanism of creativity between achievement motivation and computer-based performance, our data from the lab experiments may not be able to provide a strong causal relationship in the short term. Previous research suggests that the changeable processes between motivation influence performance in the long term (Deci & Ryan, 2000). Future studies could use the longitudinal data to explore the changeable processes of how motivation influences creativity and then changes the performance in the long term.

Our study did not consider the potential moderating effects and boundary conditions in the relationship between motivation and performance. For example, research found that individuals with higher levels of risk preferences could strengthen the relationship between motivation and performance (Nicholson et al., 2005). Thus, future studies may use experiments to explore how different moderating effects influence the effect of motivation on performance.

Finally, we did not examine how cultural differences and gender differences influence the relationship between motivation and performance in computer-based tasks. To control the potential influences of culture and gender, our study only collected data from males in China. Studies have found that male players score higher on all achievement dimensions (Yee, 2006a) and that the achievement factor is more motivational for male users (Hassouneh & Brengman, 2014). However, studies of meta-analysis found significant cultural differences and gender differences in individual motivation, creativity, and performance (Eagly & Wood, 1999; Hofstede, 2001). In the future, researchers should explore how cultural differences and gender differences affect the effect of motivation on performance.

## 6. Conclusions

Our research involved simplifying and revising the achievement motivation scale and the creativity scale, and the results indicated that the questionnaire exhibits good reliability and validity. Building on Atkinson’s (1957) achievement motivation theory to explore the mechanism of creativity within a wargaming context, we identified the impact of two dimensions of achievement motivation, hope of success and fear of failure, on wargame participants’ performance. We discovered that hope of success is significantly positively associated with performance by fostering creativity. Conversely, fear of failure demonstrates a significantly negative relationship with performance when mediated by creativity.

## Figures and Tables

**Table 1:** The KMO test and Bartlett’s test results of full scales, simplified scales, and the revised scale.

		Hope of Success	Fear of Failure	Creativity	Simplified HS	Simplified FF	Revised C
KMO test		0.928	0.944	0.955	0.631	0.649	0.823
Bartlett’s test	χ^2^	2023.053	2451.323	2346.638	121.485	189.980	585.686
	Df	105	105	78	3	3	6
	Sig	0.000	0.000	0.000	0.000	0.000	0.000

HS, hope of success; FF, fear of failure; C, creativity.

**Table 2:** Factor analysis results and reliability tests of the simplified achievement motivation scale.

Simplified Achievement Motivation Scale Items	Factor 1	Factor 2	Factor Loading	Cronbach’s α if Item Deleted	Correlation with the Total Score
I like to be persistent with problems I’m not sure I can solve.	0.619		0.817	0.602	0.799
The opportunity to be able to measure my abilities is attractive to me.	0.831		0.734	0.554	0.781
I like to do the best I can to get the job done.	0.837		0.723	0.455	0.710
I fear failure when the outcome is not known		0.818	0.842	0.605	0.831
I worry about jobs I’m not sure I can do.		0.799	0.828	0.665	0.836
The things that seem quite difficult, I worry about when I do them		0.792	0.829	0.649	0.848
Eigenvalue	2.361	1.537			
Variance explained (%)	39.343	25.614			
Cumulative variance explained (%)	39.343	64.958			

**Table 3:** Factor analysis results and reliability tests of the revised creativity scale.

Revised Creativity Scale Items	Factor 1	Factor Loading	Cronbach’s α if Item Deleted	Correlation with the Total Score
I often have novel tactical solutions during the wargame	0.862	0.844	0.837	0.854
I can come up with creative tactical strategies.	0.899	0.815	0.852	0.809
I’ll use new strategies to solve the difficulties in the wargame	0.864	0.891	0.807	0.890
I can come up with new strategies to achieve my goals	0.878	0.844	0.836	0.841
Eigenvalue	2.883			
Variance explained (%)	72.080			
Cumulative variance explained (%)	72.080			

**Table 4:** The correlation analysis between the full scales and the simplified scales.

Full Scale vs. Simplified Scales Correlation	r
Hope of success scale vs. simplified hope of success scale	0.858 **
Fear of failure scale vs. simplified fear of failure scale	0.866 **
Creativity scale vs. revised creativity scale	0.947 **

** *p* < 0.01.

**Table 5:** Goodness-of-fit values for the confirmatory factor analyses.

Model	χ²/df	GFI	CFI	TLI	IFI	RMR	RMSEA
Simplified achievement motivation	1.867	0.986	0.981	0.965	0.981	0.023	0.050
Revised creativity	4.149	0.988	0.991	0.972	0.991	0.011	0.095

df = Degrees of freedom; GFI, goodness of fit index; CFI, comparative fit index; TLI, Tucker–Lewis index; IFI, incremental fit index; RMR, root mean square residual; RMSEA, root mean square error of approximation.

**Table 6:** Mean, standard deviation, and correlation analysis results of each variable.

Variable	Mean	SD	Creativity	Hope of Success	Fear of Failure	Performance
Creativity	3.62	0.73				
Hope of Success	9.12	1.33	0.29 **			
Fear of Failure	8.09	1.51	−0.19 **	−0.11		
Performance	180.00	51.91	0.19 **	0.02	0.23 **	

** *p* < 0.01.

**Table 7:** The mediating effect.

Path	Effect	SE	Estimate	*p*	LL95%CI	UL95%CI
Hope of success → Creativity → Performance	Total effect	0.052	0.063	0.435	−0.057	0.153
	Direct effect	−0.015	0.061	0.792	−0.119	0.081
	Indirect effect	0.067	0.025	0.002	0.031	0.117
Fear of failure → Creativity → Performance	Total effect	0.235	0.071	0.003	0.111	0.344
	Direct effect	0.274	0.069	0.002	0.144	0.372
	Indirect effect	−0.040	0.022	0.012	−0.087	−0.012

5000 bootstrapped samples; 95% bootstrapped confidence intervals.

## Data Availability

The data that support the findings of this study are available from the corresponding author upon reasonable request.
